# Atypical presentation of L3 vertebral body osteochondroma mimicking cauda equina syndrome: a case report

**DOI:** 10.1097/MS9.0000000000003734

**Published:** 2025-08-15

**Authors:** Sudarsan Agarwal, Sameer Kumar Majety, Reshma Agarwal, Chandrapriya Veluru, Srinivasa Chakradhar Earni, Aakash Anumolu

**Affiliations:** a Department of Neurosurgery, Sri Venkateswara Medical College, Tirupati, India; b Department of Clinical Medicine, School of Medicine, Xiamen University, Xiamen, P.R China; c Department of Clinical Medicine, School of Medicine, China Medical University, Shenyang, P.R China; d Department of Radiation Oncology, Sri Venkateswara Medical College, Tirupati, India; e Department of Internal Medicine, International Higher School of Medicine, Bishkek, Kyrgyzstan; f Department of Internal Medicine, Bukovinian State Medical University, Chernvtsi, Ukraine

**Keywords:** benign bone tumor, partial cauda equina syndrome (CES), spinal osteochondroma, vertebral body tumor

## Abstract

**Background:**

Osteochondromas are common benign bone tumors, but spinal involvement is rare (<4%), with vertebral body origin being exceptionally uncommon. Neurological symptoms are typically mild and unilateral, making presentations mimicking incomplete cauda equina syndrome (CES) exceedingly rare.

**Case presentation:**

A 58-year-old Indian male presented with progressive lower back pain, followed by bilateral lower limb weakness and saddle anesthesia. There was no history of trauma, radiation, malignancy, or inflammatory disease. Neurological examination revealed flaccid paraplegia, areflexia, and decreased sensation below L3. Magnetic resonance imaging showed a hyperintense lytic lesion in the L3 vertebral body compressing the thecal sac; computed tomography confirmed continuity with the cortical and medullary bone, suggestive of spinal osteochondroma. The patient underwent L3 laminectomy with pedicle screw fixation at L2 and L4. Histopathology confirmed benign osteochondroma. At 6 months, the patient showed substantial motor recovery with no recurrence.

**Case discussion:**

Spinal osteochondromas usually arise from posterior elements. Vertebral body involvement is extremely rare. This case is notable for its late presentation in an elderly patient without known risk factors. The incomplete CES presentation was likely due to selective nerve root compression and possible ischemia. Imaging was critical in diagnosis, and surgical excision remains the treatment of choice. Literature supports low recurrence after complete resection, though long-term outcomes remain underreported.

**Conclusion:**

This case highlights a rare vertebral body osteochondroma mimicking incomplete CES. Early imaging and prompt surgical management are essential to avoid permanent neurological deficits. Further research is needed to understand pathogenesis in older adults without typical risk factors.

## Introduction

Osteochondroma, also known as osteocartilaginous exostosis, is the most common type of benign tumor, comprising about 10–15% of bone tumors in general^[1]^. In contrast, spinal osteochondromas constitute less than 5% of all cases. Among them, the cervical spine is most frequently involved (51%), followed by the thoracic (31%) and lumbar regions (17%)^[2]^. Most of the osteochondromas are asymptomatic and arise from the posterior vertebral column^[3]^. If symptomatic, they are primarily due to the compression of structures at their respective location. The tumors can also lead to complications such as fractures, bony deformities, mechanical joint issues, and vascular or neurological compromise^[4]^.

**HIGHLIGHTS**

- Rare vertebral body osteochondroma presenting as incomplete cauda equina syndrome.
- Elderly patient with no prior risk factors developed flaccid paraplegia and areflexia.
- Magnetic resonance imaging and computed tomography crucial in identifying lesion origin and guiding surgical intervention.
- Timely laminectomy led to neurological recovery and no recurrence at 6 months.

The pathogenesis of osteochondroma involves mutations in the EXT1 and EXT2 genes, which interfere with heparan sulfate production. This mutation halts the normal process of cartilage growth and development, leading to abnormal chondrocyte proliferation and differentiation, further causing aberrant cartilage growth and the production of osteochondromas^[4]^,

These uncommon benign lesions may induce compressive neurological symptoms, necessitating a definitive diagnosis. Imaging with a computed tomography (CT) scan is highly effective in demonstrating the maturation of the lesion in the underlying parent bone, while a magnetic resonance imaging (MRI) scan provides in-depth information about soft tissue involvement and the extent of neural involvement^[4]^. Histologically, osteochondromas show a cartilage-capped bony outgrowth continuous with the host bone’s cortex and medullary canal^[4]^.

Surgical resection remains the preferred treatment modality for symptomatic spinal osteochondromas. Complete removal of the tumor, including its cartilaginous cap, should be ensured to prevent its recurrence. Following surgical intervention, neurological symptoms often show dramatic improvement, resulting in good outcome for the patient^[3]^.

The occurrence of cauda equina-like symptoms secondary to a solitary lumbar osteochondroma is highly unusual. Only a limited number of such instances have been presented in medical literature. In this report, we present a case of a 58-year-old man who was ultimately diagnosed with a solitary osteochondroma. This case is distinct in that the lesion originated from the vertebral body of L3, mimicked incomplete cauda equina syndrome (CES), and occurred in an elderly patient without known risk factors, adding to the sparse literature on such rare spinal presentations.

## Case presentation

A 58-year-old male patient of Indian ethnicity presented with a history of gradually worsening lower back pain over several weeks, followed by progressive bilateral lower limb weakness. Over the subsequent 10 days, the weakness acutely deteriorated, resulting in complete paraplegia (Table 1). He also reported numbness in the perianal and inner thigh region, consistent with saddle anesthesia. However, he denied urinary retention, fecal incontinence, or constipation, and anal sphincter tone was preserved. There was no history of trauma, fever, unintentional weight loss, prior malignancy, or similar neurological episodes.

**Table 1:** Timeline of the clinical events

Time Point	Clinical Event
Week 4 to 2	Gradual onset of lower back pain
Day 10	Bilateral lower limb weakness began
Day 0	Presentation with paraplegia and saddle anesthesia
Day 1	MRI and CT conducted
Day 2	Surgical decompression via L3 laminectomy performed
Week 1 post-op	Histopathology confirmed osteochondroma
Month 6	Follow-up: motor improvement, persistent saddle anesthesia

On examination, the patient exhibited flaccid paralysis (Medical Research Council grade 0/5) in both lower limbs, with absent patellar and ankle reflexes, and reduced pinprick and light touch sensation below the L3 dermatome. Saddle anesthesia was confirmed, though anal tone remained normal. There was no evidence of cranial nerve involvement or upper limb abnormalities. The bladder was not distended, and there were no signs of bowel dysfunction at the time of evaluation.

Given the clinical features of acute paraplegia with saddle anesthesia, a space-occupying lesion causing spinal cord or cauda equina compression was suspected. A provisional diagnosis of atypical or incomplete CES secondary to a compressive lesion at the L3 level was made, with differentials including primary or metastatic spinal tumor.

**Figure 1.**
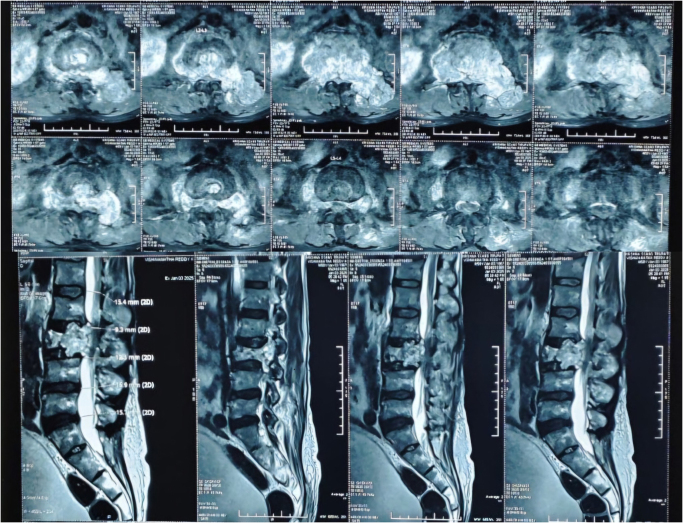
MRI scans showing axial (top) and sagittal (bottom) views of the lumbar spine. A bony mass is visible, likely indicating a lesion compressing the spinal canal. MRI, magnetic resonance imaging.

**Figure 2.**
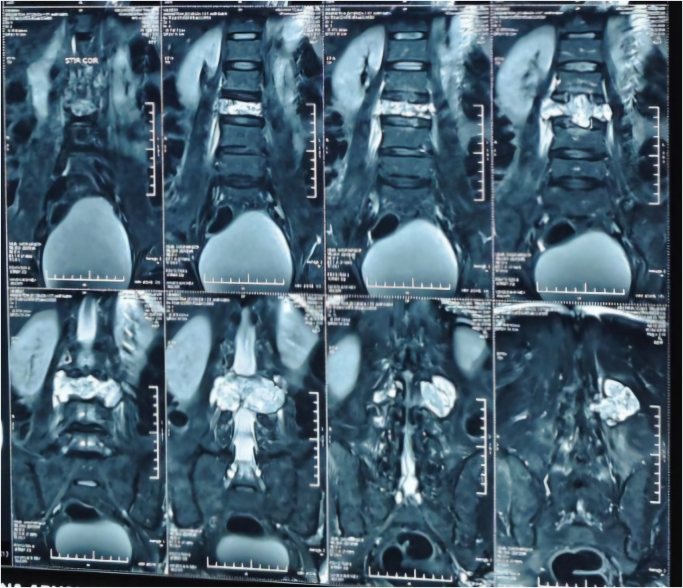
MRI coronal views of the lumbar spine and pelvis showing a heterogeneous bony mass arising near the vertebrae, displacing adjacent soft tissues. The lesion appears lobulated and dense, suggesting a possible osteogenic origin. MRI, magnetic resonance imaging.

Magnetic resonance imaging of the lumbar spine revealed a hyperintense, irregular, lytic lesion at the L3 vertebral body, causing posterior displacement of the thecal sac and myelographic cutoff at the L3 level.(Figs. 1 and 2) The lesion extended into adjacent intervertebral discs, causing significant spinal canal compromise. Computed tomography further demonstrated a bony exostosis continuous with the cortex and medullary cavity of the L3 vertebra, resulting in canal narrowing and displacement of neural elements. These findings were consistent with a diagnosis of spinal osteochondroma.

**Figure 3.**
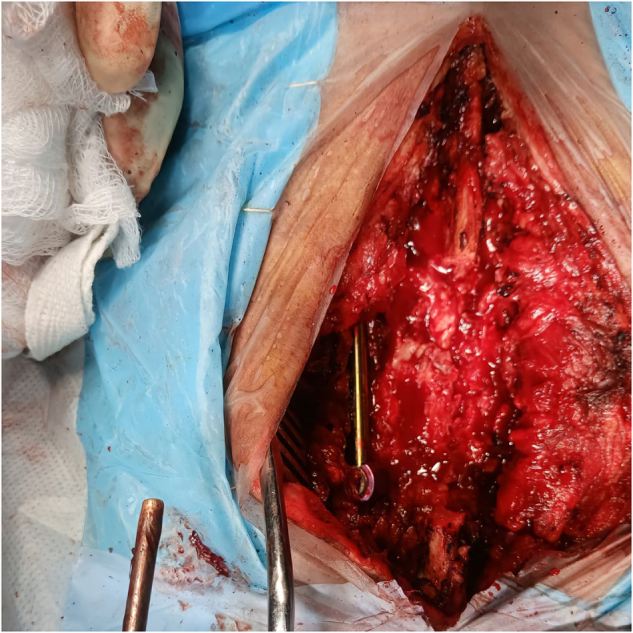
Intraoperative image showing an exposed spinal surgical field with a visible pedicle screw and rod implant. Retractors hold back soft tissue, revealing underlying vertebral structures and active dissection.

The patient underwent surgical decompression via L3 laminectomy with instrumented stabilization using pedicle screws at L2 and L4.(Fig. 3) The lesion was partially excised and sent for histopathological evaluation. General anesthesia was administered using a standard balanced technique. Preoperative laboratory investigations (complete blood count [CBC[, renal and liver function tests, electrolytes, C-reactive protein [CRP]) were within normal limits. Intraoperative prophylactic antibiotics (cefazolin) and postoperative analgesics (paracetamol and tramadol) were administered as per institutional protocol. Intraoperatively, mild bleeding was encountered and managed appropriately. The procedure was completed uneventfully. There were no immediate postoperative complications, and the patient was monitored regularly during the recovery period.

Histopathological analysis of the excised specimen revealed mature bony trabeculae with osteoblastic rimming, an overlying hyaline cartilage cap with focal calcification, and areas of fibrosis and hemorrhage. No evidence of malignancy was seen, confirming the diagnosis of a benign osteochondroma.

At 6-month follow-up, the patient demonstrated gradual improvement in lower limb strength, with preserved bowel and bladder function, though saddle anesthesia persisted. He remained under active physiotherapy with good functional recovery, and follow-up imaging showed no signs of recurrence.

This case has been reported in line with the scare 2025 criteria^[5]^.

## Case discussion

### Overview and epidemiology

Osteochondroma represents the most frequently occurring benign tumor of bone, accounting for up to 30–40% of all benign bone tumors^[6]^. It typically manifests as a cartilage-capped osseous outgrowth arising from the metaphyseal regions of long bones such as the femur, tibia, and humerus and contains marrow cavity continuous with underlying bone^[7]^. Several studies report a notable male predominance in its occurrence^[6]^.

While osteochondromas frequently occur in the appendicular skeleton, their presence in the spine is rare, representing only about 1.2% of cases. When spinal involvement occurs, the cervical region is most commonly affected, accounting for around 50%^[7,8]^ Mardi *et al* suggest that secondary ossification centers appear earlier in the cervical-thoracic spine, around age 11, compared to approximately age 18 in the lumbar spine. This earlier development may predispose the cervical spine to aberrant cartilage growth, particularly when ossification occurs rapidly^[9]^.

Among spinal osteochondromas, the majority originate from the posterior elements including the lamina, spinous, transverse, or articular processes, due to their proximity to secondary ossification centers^[7,8]^. Vertebral body involvement, as observed in the current case, remains exceptionally uncommon and sparsely documented in the literature^[8]^.

### Pathophysiology

These tumors can manifest as solitary lesions or as part of multiple hereditary exostoses, which occurs in an autosomal dominant fashion^[7]^. Unlike in this case, symptoms of cord compression have been reported to occur twice as frequently in MHE cases^[6,9]^, making this presentation unique. Despite being benign in nature, malignant transformation is seen in approximately 1% of solitary osteochondromas and in 3–5% of patients with MHE^[10]^.

Osteochondroma may also occur in patients with radiation exposure in childhood. These radiation-induced lesions tend to be pathologically and radiologically indistinguishable from spontaneously occurring solitary osteochondroma or in hereditary multiple exostoses^[11]^. Radiation-induced osteochondroma are extremely rare in lumbar spine^[8]^.

In elderly patients, lumbar osteochondromas are also rare and might be related to degenerative changes in the vertebrae that promote abnormal cartilage growth^[9,12]^. This might be due to the higher prevalence of chronic inflammatory conditions such as psoriatic arthritis in older adults, where the associated accelerated bone metabolism and abnormal cartilage growth may predispose them to develop osteochondromas even after skeletal maturity^[9]^. Stevens *et al* reported chondroid metaplasia in paraspinal connective tissue in the context of spinal degeneration, presumably as a result of local inflammation and aberrant mesenchymal progenitor behavior. This chondrocytic metaplasia, with time, might predispose to neoplastic change. This aberrant differentiation might have the potential of developing into benign cartilaginous tumor such as osteochondroma, particularly under chronic inflammatory or degenerative stimuli^[13]^.

Our case is distinct in that the patient presented symptomatically at the age of 58, with no history of childhood radiation, trauma, or chronic inflammatory conditions such as psoriatic arthritis. Genetic testing for MHE was not pursued, as the patient had no family history of exostoses, no evidence of multiple lesions on imaging, and no phenotypic features suggestive of hereditary syndromes. This highlights the need for a deeper understanding of the underlying pathophysiology in elderly patients with osteochondroma.

### Mechanism of CES

Cauda equina syndrome is a rare but emergent neurologic condition arising due to lumbosacral nerve root compression distal to the conus medullaris. Classic features include bilateral lower extremity weakness, saddle anesthesia, bladder and bowel dysfunction, and sexual dysfunction, only some of which were evident in this patient^[14]^.

The mechanism by which CES occurred in this patient is that of a space-occupying lesion compressing the cauda equina nerve roots. In our patient, saddle anesthesia occurred and persisted without bladder or bowel involvement, and is presumed due to the medial location of the lower sacral (S2–S5) sensory fibers. A midline lumbar invlovement can selectively compress these roots while sparing the motor fibers that are responsible for bladder and bowel function^[15,16]^.

Imaging showed the presence of an exophytic bony mass arising from the posterior aspect of the L3 vertebral body, with osteochondroma being the leading differential diagnosis. The anterior nerve roots undergo compression in CES, giving rise to lower motor neuron signs, flaccid paralysis, areflexia, and autonomic dysfunction due to disruption of the peripheral motor and sensory pathways^[15]^. In this case, MRI and histopathology confirmed a benign L3 osteochondroma causing thecal sac compression and spinal canal narrowing.

Delay in managing CES causes permanent neurologic sequelae such as chronic incontinence, persistent sexual dysfunction, and paraplegia^[17]^. Only one article has described a potential mechanism for partial CES. This study stated that it might be due to mechanical compression and a possible ischemia due to venous congestion. In our case, the exact underlying mechanism remains unclear and warrants further investigation through studies with a broader scope. Further investigation into the role of inflammatory mediators, vascular compromise, and individual anatomical variations may provide insights into such atypical presentations^[18]^.

### Diagnostics and differentials

The role of imaging in a solitary osteochondroma can be invaluable. Even though a plain radiograph might miss early lesions, advanced techniques like a CT scan or an MRI scan provide vital information. A CT scan usually shows continuity between the cortical and medullary aspects of the underlying bone and the mass. A case series studying five lumbosacral osteochondromas have reported that all of the five cases have shown a honey-comb pattern at the center of the tumor. The cancellous central portion and the associated cartilaginous cap appear as high-intensity signals on T2-weighted MRI^[19]^. An MRI scan also provides crucial information in cases of thecal sac or nerve impingement, epidural extension, and secondary changes such as edema or bursitis. Additionally, if the thickness of the cartilaginous cap exceeds 20 mm, it might raise suspicion for chondrosarcoma^[20]^. In our case, CT showed cortical and medullary continuity, and MRI revealed neural compression, findings consistent with typical imaging characteristics of spinal osteochondroma.

The other differentials that can be considered are osteophytes, osteoblastomas, chondrosarcomas, and meningiomas. A key diagnostic factor is continuity of the cortex and marrow between the lesion and the parent vertebra. Histologically, osteochondroma might appear as trabecular bone capped by cartilage and usually done postoperatively to rule out malignancy. Signs of an underlying malignancy might include but not be limited to cap >20 mm, irregular margins, and marrow infiltration^[20]^.

In our patient, inflammatory markers and autoimmune panels were not pursued, as there were no systemic symptoms such as fever or weight loss. Additionally, baseline laboratory parameters, including CBC, renal and liver function tests, electrolytes, and CRP, were within normal limits. Imaging and histopathology were sufficient to confirm a benign, non-inflammatory etiology.

### Management and prognosis

Surgical excision is the mainstay of treatment in case of a symptomatic spinal osteochondroma. Complete removal of the mass including the cap is advised to alleviate the compressive symptoms and prevent the recurrence^[21]^.Similar to our case, laminectomy, hemilaminectomy, or facetectomy at the involved level is typically performed using a posterior surgical approach. Two different reviews each with 27^[21]^ and 35^[22]^ lumbar osteochondromas respectively have confirmed the same.

A study by Yakkanti *et al*, which has a sample size of more than 40 symptomatic osteochondromas, has stated that the rate of recurrence is 0% among spinal osteochondromas with myelopathic symptoms. However, a study focused on long-term outcomes in primary spinal osteochondromas reported that recurrence rates are as high as 8% (2 out of 27 patients)^[23]^. The contrasting nature of these findings may be due to the smaller sample size of the latter study, lack of long-term follow-up, or possible underreporting of recurrences in the former study.

In our case, the patient showed no signs of recurrence at the 6-month follow-up visit. However, this duration is insufficient to fully assess long-term outcomes. As this is a recent case, longer follow-up has not yet been completed. The patient was advised to attend monthly follow-up.

## Limitations

This case report is limited by its single-subject design, which restricts the generalizability of the findings. However, it provides a valuable foundation for future studies exploring the clinical spectrum and management of spinal osteochondromas in elderly patients. The 6-month follow-up period may be insufficient to fully assess late recurrence, long-term functional outcomes, or delayed complications. Longer-term data could not be obtained at this time as this is a recent case, and the patient remains under active surveillance.

The etiology of the cauda equina-like symptoms without bladder or bowel dysfunction remains speculative, as there was no intraoperative or histological evidence of ischemia or vascular compromise. Furthermore, the absence of genetic testing for EXT1/EXT2 mutations and inflammatory marker analysis limits a more detailed understanding of the tumor’s pathogenesis. These gaps underscore the need for prospective studies investigating the role of genetic and inflammatory mechanisms, as well as vascular and anatomical factors, in atypical presentations of spinal osteochondroma.

## Conclusion

This case presents an unusual presentation of spinal osteochondroma in a 58-year-old patient without a history of trauma, childhood irradiation, or long-term inflammatory diseases like psoriatic arthritis. It emphasizes the need for a deeper understanding of the pathophysiological mechanisms underlying osteochondroma development in older adults, particularly in the absence of typical risk factors. Clinicians must have a high suspicion of spinal osteochondroma in the assessment of partial or incomplete CES, particularly in patients with progressive back pain and subacute or sudden-onset neurological deficits. Early diagnosis and prompt surgical intervention are essential to avoid permanent neurological damage and maximize functional recovery. Additional studies are required to investigate genetic or microenvironmental causes of late-onset spinal osteochondroma. Extended clinical and radiological surveillance is recommended, given the potential for delayed recurrence. Future studies should investigate the genetic, degenerative, and microenvironmental factors that may contribute to the development of late-onset spinal osteochondromas, especially in patients lacking classical predisposing conditions.

## Data Availability

The data regarding the patient can be submitted by the corresponding author upon reasonable request.

## Research registration unique identifying number (UIN)

This article is a case report and is therefore exempt from registration in a public research registry, in accordance with the policies of the *Annals of Medicine and Surgery*.

## References

[1] Murphey MD Choi JJ Kransdorf MJ. Imaging of osteochondroma: variants and complications with radiologic-pathologic correlation. Radiographics 2000;20:1407–34. 10992031 10.1148/radiographics.20.5.g00se171407

[2] Gille O Pointillart V Vital JM. Solitary spinal osteochondromas: a review of 59 cases. Spine (Phila Pa 1976) 2005;30:E35–E40. 15626967

[3] Sinelnikov A Kale H. Osteochondromas of the spine. Clin Radiol 2014;69:e584–e590. 25282617 10.1016/j.crad.2014.08.017

[4] Kitsoulis P Galani V Stefanaki K. Osteochondromas: review of the clinical, radiological and pathological features. In Vivo 2008;22:633–46. 18853760

[5] Kerwan A Al-Jabir A Mathew G. Revised Surgical CAse REport (SCARE) guideline: an update for the age of Artificial Intelligence. Premier J Sci 2025;10:100079.

[6] Albrecht S Crutchfield JS SeGall GK. On spinal osteochondromas. J Neurosurg 1992;77:247–52. 1625013 10.3171/jns.1992.77.2.0247

[7] Shigekiyo S Nishisho T Takata Y. Intracanalicular osteochondroma in the lumbar spine. NMC Case Rep J 2019;7:11–15. 31938676 10.2176/nmccrj.cr.2019-0031 PMC6957774

[8] Suwak P Barnett SA Song BM. Atypical osteochondroma of the lumbar spine associated with suprasellar pineal germinoma: a case report. World J Orthop 2021;12:720–26. 34631455 10.5312/wjo.v12.i9.720 PMC8472447

[9] Mardi K Madan S. Pediatric solitary osteochondroma of T1 vertebra causing spinal cord compression: a case report. South Asian J Cancer 2013;2:144. 10.4103/2278-330X.114130 PMC3892549 24455595

[10] Ramdasi RV Mahore A. Solitary thoracic osteochondroma presenting as Brown-Séquard syndrome. BMJ Case Rep 2014;2014:bcr2014206656. 10.1136/bcr-2014-206656 PMC4244355 25404250

[11] Gorospe L Madrid-Muñiz C Royo A. Radiation-induced osteochondroma of the T4 vertebra causing spinal cord compression. Eur Radiol 2002;12:844–48. 11960236 10.1007/s003300101034

[12] Sakai D Mochida J Toh E. Spinal osteochondromas in middle-aged to elderly patients. Spine (Phila Pa 1976) 2002;27:E503–E506. 12461407 10.1097/00007632-200212010-00017

[13] Stevens S Agten A Wisanto E. Chondroid metaplasia of paraspinal connective tissue in the degenerative spine. Anat Cell Biol 2019;52:204–07. 31338238 10.5115/acb.2019.52.2.204 PMC6624339

[14] Rider LS Marra EM. Cauda equina and conus medullaris syndromes. StatPearls [Internet]. Treasure Island (FL):StatPearls Publishing; 2023. Accessed 19 August 2025. https://www.ncbi.nlm.nih.gov/books/NBK537200/30725885

[15] Orendácová J Cízková D Kafka J. Cauda equina syndrome. Prog Neurobiol 2001;64:613–37. 11311464 10.1016/s0301-0082(00)00065-4

[16] Bang JH Cho KT. Missed cauda equina syndrome after burst fracture of the lumbar spine. Korean J Neurotrauma 2015;11:175–79. 27169089 10.13004/kjnt.2015.11.2.175 PMC4847493

[17] Gitelman A Hishmeh S Morelli BN. Cauda equina syndrome: a comprehensive review. Am J Orthop 2008;37:556–62. 19104682

[18] Vitelli M Barbagli G Peppucci E. A rare case of partial cauda equina syndrome following decompression for spinal stenosis: an illustrative case. Arq Bras Neurocir 2025;44:e47–e50.

[19] Kuraishi K Hanakita J Takahashi T. Symptomatic osteochondroma of lumbosacral spine: report of 5 cases. Neurol Med Chir (Tokyo) 2014;54:408–12. 24172589 10.2176/nmc.cr2012-0049 PMC4533430

[20] Bernard SA Murphey MD Flemming DJ. Improved differentiation of benign osteochondromas from secondary chondrosarcomas with standardized measurement of cartilage cap at CT and MR imaging. Radiology 2010;255:857–65. 20392983 10.1148/radiol.10082120

[21] Yakkanti R Onyekwelu I Carreon LY. Solitary osteochondroma of the spine—A case series: review of solitary osteochondroma with myelopathic symptoms. Global Spine J 2018;8:323–39. 29977716 10.1177/2192568217701096 PMC6022963

[22] Kahveci R Ergüngör MF Günaydın A. Lumbar solitary osteochondroma presenting with cauda equina syndrome: a case report. Acta Orthop Traumatol Turc 2012;46:468–72. 23428773 10.3944/aott.2012.2599

[23] Sciubba DM Macki M Bydon M. Long-term outcomes in primary spinal osteochondroma: a multicenter study of 27 patients. J Neurol Neurosurg Spine 2015;22:582–88. 10.3171/2014.10.SPINE14501 PMC4859212 25793467

